# Trunk muscle fatigue during a lateral isometric hold test: what are we evaluating?

**DOI:** 10.1186/2045-709X-20-12

**Published:** 2012-04-19

**Authors:** Isabelle Pagé, Martin Descarreaux

**Affiliations:** 1 Département des sciences de l’activité physique, Université du Québec à Trois-Rivières, Trois-Rivières, G9A 5H7, Canada; 2 Département de chiropratique, Université du Québec à Trois-Rivières, Trois-Rivières, G9A 5H7, Canada

**Keywords:** Side bridge test, Muscle fatigue, Trunk muscle, Functional testing

## Abstract

**Background:**

Side bridge endurance protocols have been suggested to evaluate lateral trunk flexor and/or spine stabilizer muscles. To date, no study has investigated muscle recruitment and fatigability during these protocols. Therefore the purpose of our study was to quantify fatigue parameters in various trunk muscles during a modified side bridge endurance task (i.e. a lateral isometric hold test on a 45° roman chair apparatus) and determine which primary trunk muscles get fatigued during this task. It was hypothesized that the ipsilateral external oblique and lumbar erector spinae muscles will exhibit the highest fatigue indices.

**Methods:**

Twenty-two healthy subjects participated in this study. The experimental session included left and right lateral isometric hold tasks preceded and followed by 3 maximal voluntary contractions in the same position. Surface electromyography (EMG) recordings were obtained bilaterally from the external oblique, rectus abdominis, and L2 and L5 erector spinae. Statistical analysis were conducted to compare the right and left maximal voluntary contractions (MVC), surface EMG activities, right vs. left holding times and decay rate of the median frequency as the percent change from the initial value (NMF_slope_).

**Results:**

No significant left and right lateral isometric hold tests differences were observed neither for holding times (97.2 ± 21.5 sec and 96.7 ± 24.9 sec respectively) nor for pre and post fatigue root mean square during MVCs. However, participants showed significant decreases of MVCs between pre and post fatigue measurements for both the left and right lateral isometric hold tests. Statistical analysis showed that a significantly NMF_slope_ of the ipsilateral external oblique during both conditions, and a NMF_slope_ of the contralateral L5 erector spinae during the left lateral isometric hold test were steeper than those of the other side’s respective muscles. Although some participants presented positive NMF_slope_ for some muscles, each muscle presented a mean negative NMF_slope_ significantly different from 0.

**Conclusions:**

Although the fatigue indices suggest that the ipsilateral external oblique and contralateral L5 erector spinae show signs of muscle fatigue, this task seems to recruit a large group of trunk muscles. Clinicians should not view this task as evaluating specifically lateral trunk flexors, but rather as providing an indication of the general endurance and stabilisation capacity of the trunk.

## Background

Over the past two decades, lumbar spine stability has become an integral part of the low back pain assessment and treatment strategies, especially given its potential link to injury mechanisms and the ongoing clinical efforts directed toward enhancing stability in patients [1]. Furthermore, an increasing number of researchers and clinicians consider the strategy used by patients to activate their abdominal muscles to be central to the stability theme. It has been demonstrated that bracing, defined as the increase of torso stiffness by the activation of all abdominal muscles and back extensors muscles, produces greater stability than hollowing, which consist of the activation of the transversus abdominis and internal oblique muscles in healthy subjects [1,2]. As a corollary, a variety of trunk coactivation exercise protocols are frequently used in daily clinical practice for low back pain prevention in healthy patients, rehabilitation in low back pain patients, or in order to evaluate trunk muscle function. The quadratus lumborum, external oblique, internal oblique, iliocostalis, longissimus and intertransversalis are believed to act as spine stabilizers when contracting bilaterally and as lateral trunk flexors by pulling the rib cage toward the hip when contracting unilaterally [3].

McGill et al. [4] suggested that a difference between left and right endurance time of the trunk flexors, extensors and lateral flexors muscles would predict who is at greater risk of back problem. As no exercise can evaluate all muscles involved in lumbar spine stability, evaluation in the 3 planes has to be done separately. Side bridge exercise protocols have been suggested to evaluate torso muscles in frontal plane. Such exercises are usually executed in a position where the participant lies down sideways with support of one arm and are named after the side of the arm support (e.g. left side bridge = left arm support) [5] Variants to the protocol initially described by McGill have also been described [5-7].

A wide range of use of these exercises has been presented in the literature. Some authors proposed comparing muscle balance by evaluating holding times, whereas others assessed the use of maximal voluntary contraction or isometric contraction of short duration to evaluate muscle recruitment.

McGill et al. [8] evaluated the holding times of 75 healthy subjects (mean age of 23 years) during side bridge protocols and obtained mean times of 81 ± 34 sec and 85 ± 36 sec for right and left side bridge respectively. Other authors [9] reported similar mean times, i.e. 87.5 ± 36.4 sec and 92.0 ± 45.8 sec for right and left side bridges respectively, in a group of 24 healthy subjects (mean age of 35.3 years). McGill [10] has proposed that endurance scores during side bridges could be interpreted by using a right side to left side holding time ratio. A discrepancy of over 0.05 in the ratio would suggest unbalanced endurance. McGill et al. [8] also examined the intra-rater reliability of this test in 5 subjects on 5 consecutive days and at 8 weeks (follow-up) and got an excellent reliability coefficient of over .96. Evans et al. [9] reported high intra-rater and inter-rater reliability with the lowest coefficient being .81 and .82 respectively.A number of studies have also provided information about muscle recruitment during side bridge tests. McGill et al. [11] reported ipsilateral (left side during side bridges with left supporting arm) trunk muscle activation of the quadratus lumborum, external oblique, rectus abdominis and lumbar erector spinae during isometric side bridges. These muscles presented 54%, 40%, 22% and 24% of their maximal voluntary contraction activity (MVCA) respectively. However, the small sample (4 participants) does not allow for much generalization. Ekstrom et al. [12] evaluated muscle activation during 5-second hold side bridges and reported an activation of up to 72% of MVCA of the gluteus medius, 69% of the external oblique, between 34% and 42% of the longissimus thoracis, lumbar multifidus and rectus abdominis and less of 21% of the gluteus maximus and harmstring muscles. In 2008, Ekstrom et al. [13] used the same protocol to evaluate the longissimus thoracis and lumbar multifidus activation on both sides during side bridges. The authors reported greater activation of the ipsilateral longissimus thoracis than of the lumbar multifidus (48-49% vs. 32-33% of MVCA respectively) and greater activation on the ipsilateral side than on the contralateral side (48-49% vs 7-8% of MVCA for the longissimus thoracis and 32-33% vs 14% of MVCA for the lumbar multifidus). They also evaluated muscle activation during maximal resistance in side bridge without trunk support (legs fixed and trunk unsupported) and reported greater activation of the ipsilateral (left muscle when left side up) longissimus thoracis at L1 than of the lumbar multifidus (54-58% and 38-39% of MVCA respectively). They demonstrated a greater activation on the ipsilateral side than on the contralateral one (54-58% vs. 7-9% of MVCA for longissimus thoracis and 38-39% vs. 12-13% of MVCA for lumbar multifidus). In light of these studies, the external oblique, gluteus medius and back extensor muscles on the ipsilateral side of the side bridge seem to be the muscles with the greatest levels of activation during this exercise.

Although muscle activation during side bridge endurance and trunk lateral flexion has been described in a few studies, muscle recruitment and fatigability protocols have not been extensively studied. Therefore the purpose of our study was to quantify fatigue parameters in various trunk muscles during a modified side bridge endurance test (i.e. a lateral isometric hold test on a 45° roman chair apparatus). A second objective was to determine which primary trunk muscles are fatigued during this functional task. We hypothesized that the ipsilateral external oblique and erector spinae muscles would exhibit the highest fatigue indices during the modified side bridge protocol.

## Methods

### Participants

Twenty-two healthy subjects (11 men, 11 women; mean age ± SD: 24.55 ± 5.00) participated in this study. All participants were volunteers and gave their informed, written consent according to the protocol approved by the Université du Québec à Trois-Rivières (Canada) Ethics Committee.

### Experimental protocol

The one hour experimental session included maximal voluntary contractions and a trunk muscle endurance task in a lateral isometric hold position. Participants’ height and weight were measured prior to the experimental task. The *Baecke-f* questionnaire was also completed by all participants in order to assess their physical activity levels (daily participation in sports and leisure activity) [14]. The experimental task was thoroughly explained and demonstrated by the experimenter before any data were recorded.

### Maximal voluntary contraction

The endurance task was preceded and followed by three maximal voluntary contractions (MVC) in the same position, and participants were allowed a 2-minute rest period between the MVC pre-endurance and endurance task, and 15 minutes between post-endurance MVC and the other side pre-endurance MVC. MVC assessments, conducted 2 minutes prior to, and immediately after each endurance protocol, were performed against a force transducer that measured trunk muscle strength in the lateral position in accordance with the procedure presented by Ledoux et al. [7]. Participants were tested in the same position they held in the endurance tasks. Verbal encouragements were provided to maximize voluntary contraction. MVCs were performed against a fixed harness around the shoulders connected inline to a uni-axial force transducer on the floor (NTEP-87-057A3 class III, Artech, Riverside, CA, USA). For each MVC trial, force data were recorded at a sampling rate of 1,000 Hz and filtered digitally with a fifth-order Butterworth filter (10-Hz low-pass cutoff frequency). The higher force value obtained in 3 consecutive 10-s trials was used as the reference for MVC.

### Trunk muscle endurance task

**Figure 1:**
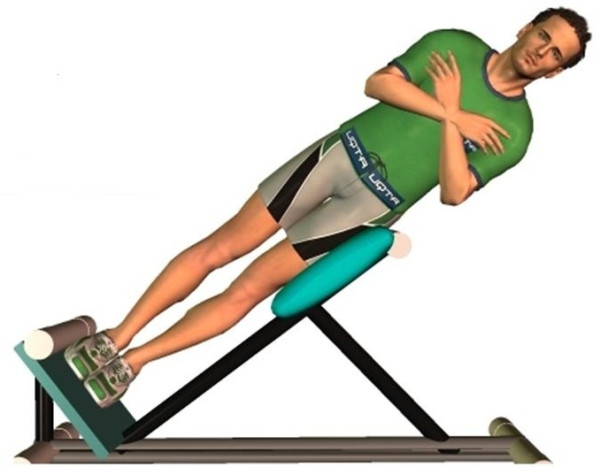
Apparatus and participants’ positioning for right lateral isometric hold test.

Participants were asked to perform sustained isometric contractions of the trunk muscles in a lateral position for both left and right sides named according to the side up (Figure 1). The endurance tasks (left and right) were counterbalanced across participants to control for sequence order effects. The protocol was explained and demonstrated before any experimental task was undertaken. During the left lateral isometric hold test, participants were positioned on the right side on a 45 degrees Roman chair. Ledoux et al. [7] suggested that this protocol could be used as an alternative to the side bridge test. When used to assess muscle fatigability, it yielded endurance time slightly longer than to those reported by McGill[10]. The test was developed as an alternative to the side bridge test to evaluate endurance time in older adults and adults with upper limb injuries (who could not attain support off the floor). This test also creates the possibility to assess maximal voluntary contraction in the same position. The trunk, from the anterior superior iliac spine and up, was unsupported. Arm support was allowed prior to the endurance task. On the researcher’s cue, the participants removed their arms from the support and folded them across the chest with hands placed on the opposite shoulder. During the endurance task, participants were asked to keep their trunk and head in line with their lower limbs which were one above the other. The goal for all participants was to hold this position as long as they could. In order to ensure that participants abide by instructions, the same assistant observed the entire task for every participant and gave them verbal feedback to ensure proper position based on thorough observation. Failure to comply with instructions resulted in a warning by the assistant and the task was ended if the participant failed to follow instructions three times. Verbalized encouragement was provided throughout the test.

### Electromyography (EMG)

Surface EMG data were collected with *BiPole* disposable surface Ag-AgCl electrodes (Bortec biomedical, Calgary, Alberta, Canada) applied bilaterally on specific muscles in line with fiber direction. Inter-electrode distance was fixed at 2 cm and electrode diameter was 1 cm. Muscle activity of the external oblique, rectus abdominis, lumbar erector spinae (L2 and L5 level) was recorded according to McGill et al. [15]. A ground electrode was placed on the left olecranon of each participant. Skin impedance was reduced by (1) shaving body hair, (2) gently abrading the skin with fine-grade sandpaper (Red Dot Trace Prep, 3 M, St. Paul, MN, USA), and (3) wiping the skin with alcohol swabs. EMG activity was recorded using a single differential Delsys Surface EMG sensor with a common mode rejection ratio of 92 dB at 60 Hz, a noise level of 1.2 μV, a gain of 10 v/v ± 1%, an input impedance of 10^15^ Ω (Model DE2.1, Delsys Inc., Boston, MA, USA) and sampled at 1,000 Hz with a 12-bit A/D converter (PCI 6024E, National Instruments, Austin, TX, USA). The EMG data were filtered digitally by a 10- to 450-Hz band-pass, zero-lag, fifth-order Butterworth filter. They were collected by LabView (National Instruments) and processed by Matlab (MathWorks, Natick, MA, USA).

### Data analysis

Maximal voluntary contraction (Newton) and maximal EMG root mean square values (RMS) (for each muscle) were obtained for every MVC trial to assess muscle fatigue. Muscular fatigue was assessed during the fatigue protocol through power spectral analysis. Median power frequency (MedF) was calculated from successive non-overlapping windows of 250 ms by Fast-Fourier transformation. Least square linear regression analysis was applied to MedF time series (MedF as a function of time) to estimate the rate of decline (MedF_slope_). In order to express the decay rate of MedF as the percent change from the initial value (NMF_slope_ in%s^-1^), MedF_slope_ were divided by the initial MedF [16,17] . The equation below was used to obtained individual holding time ratios:

(1) $$\left|\;1-\left(\text{right holding time }/\text{ left holding time}\right)\;\right|$$

### Statistical analysis

Right and left maximal voluntary contractions (Newtons) and RMS were compared between pre and post endurance tasks with 2-tailed *t*-tests for dependent samples. 2-tailed *t*-tests for dependant samples were also used to compare holding times between left and right lateral isometric hold tests. In order to test for our main hypothesis, the NMF_slope_ of antagonist muscles in both conditions (e.g. left external oblique vs. right external oblique during left lateral isometric hold test) were compared using 2-tailed *t*-tests for independent samples. *T*-tests for independent samples (1-tailed) comparing NMF_slope_ to 0 were conducted for every muscle in both conditions to determine if statistically significant decay of NMF were induced by the fatigue protocols. Simple correlation tests were performed separately for each baseline characteristic and holding times in order to evaluated the possible correlations. Statistical significance was set at *p* ≪ 0.05 for all analyses.

## Results

### Participants’ characteristics

Table 1 reports the mean (SD) baseline characteristics of participants (means age, weight, height, body mass index, Baecke-f questionnaire score).

**Table 1:** Participants’ characteristics (mean ± SD)

	Mean ± SD
Age (years)	24.55 ± 5.00
Weight (kg)	70.62 ± 8.86
Height (m)	1.72 ± 0.09
Body mass index (kg/m^2^)	23.94 ± 2.78
Sport activity indices in Baecke-f questionnaire	5.40 ± 1.51
Free-time activity indices in Baecke-f questionnaire	3.06 ± 0.79

### Holding times and maximal voluntary contractions

**Figure 2:**
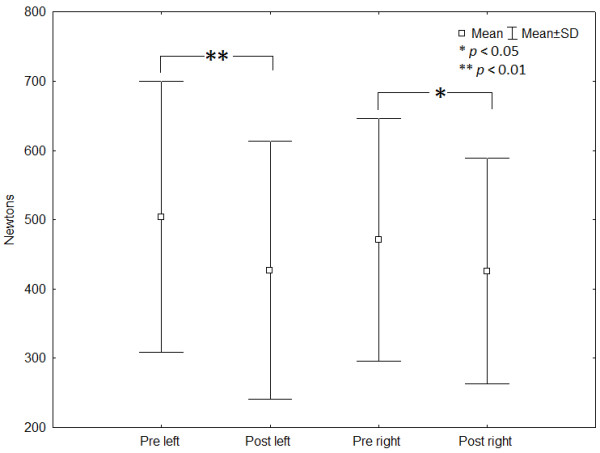
Left and right lateral isometric hold test MVCs before and after the endurance task.

*T*-tests for dependant samples revealed no significant difference between sides for holding times (*p* ≫ 0.88) but showed significant decreases of MVCs between pre and post endurance task measurements for both the left and right lateral isometric hold tests (*p* ≪ 0.01 and *p* = 0.02 respectively). No significant differences were observed between RMS pre and post endurance task values (*p* ≫ 0.05). The calculated mean individual holding time ratio showed a 0.119 ± 0.118 discrepancy between the right and left sides. Simple correlation tests revealed no significant correlation between holding times and each baseline characteristic (*p* ≫ 0.05). Figure 2 and Table 2 present the mean ± standard deviation for holding times and MVC statistical analysis.

**Table 2:** Left and right lateral isometric hold tests holding times and ratios (mean ± SD)

	Mean ± SD
Left lateral isometric hold test holding times (sec)	96.7 ± 24.9
Right lateral isometric hold test holding times (sec)	97.2 ± 21.5
Holding time ratio	0.119 ± 0.118
*P* value (between left and right lateral isometric hold tests)	0.88

### Muscles recruitment during endurance task and muscle fatigue indices

**Figure 3:**
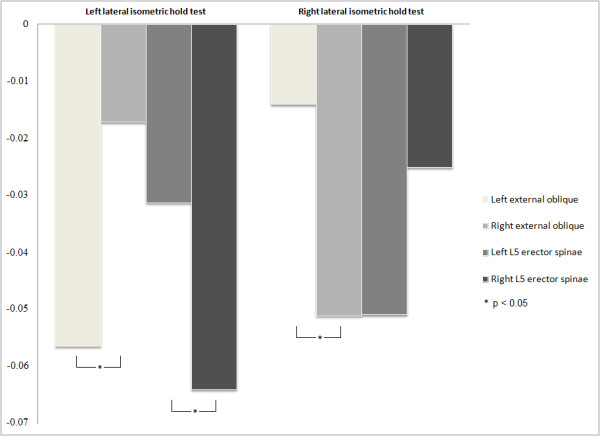
**NMF**_**slope**_**(%s**^**-1**^**) of external oblique and L5 erector spinae muscles during left and right lateral isometric hold tests.**

Figure 3 presents the NMF_slope_ of external oblique and L5 erector spinae muscles for both sides during left and right lateral isometric hold tests. *T*-tests for independent samples comparing NMF_slope_ of antagonist muscles in both conditions (e.g. left external oblique vs. right external oblique during left lateral isometric hold test) showed a significantly greater decay rate of the NMF_slope_ of ipsilateral external oblique (i.e. left external oblique while left lateral isometric hold test comparing to right external oblique) during both conditions and of right L5 erector spinae during left lateral isometric hold test comparing to the left muscle. During right lateral isometric hold test, the left L5 erector spinae presented a trend toward a greater decay rate of the NMF_slope_ than the right one, but these differences were not significant (*p* = 0.08). No difference was observed in the other left versus right muscle comparisons (L2 erector spinae and rectus abdominis) in any of the conditions (*p* ≫ 0.05). Table 3 presents results of *t*-tests for independent samples for NMF_slope_ of each muscle recorded during both conditions. Although some participants presented positive NMF_slope_ for some muscles, each muscle presented a mean negative NMF_slope_ significantly different from 0 at *p* ≪ 0.05.

**Table 3:** **NMF**_**slope**_***t*-tests for left and right lateral isometric hold tests (mean ± SD)**

**Muscles**	**Left lateral isometric hold test**			**Right lateral isometric hold test**		
	df	NMF_slope_	*P*	df	NMF_slope_	*P*
		(% s^-1^)	values		(% s^-1^)	values
Left rectus abdominis	19	−0.025 ± 0.054	0.018	20	−0.037 ± 0.043	≪ 0.001
Right rectus abdominis	18	−0.032 ± 0.038	≪ 0.001	20	−0.026 ± 0.023	≪ 0.001
Left external oblique	18	−0.056 ± 0.037	≪ 0.001	20	−0.014 ± 0.026	0.0106
Right external oblique	19	−0.017 ± 0.030	0.009	20	−0.051 ± 0.039	≪ 0.001
Left L2 erector spinae	20	−0.023 ± 0.031	≪ 0.001	20	−0.034 ± 0.054	0.0041
Right L2 erector spinae	20	−0.043 ± 0.051	≪ 0.001	20	−0.025 ± 0.029	≪ 0.001
Left L5 erector spinae	20	−0.031 ± 0.053	0.0011	17	−0.051 ± 0.031	≪ 0.001
Right L5 erector spinae	18	−0.064 ± 0.048	≪ 0.001	20	−0.025 ± 0. 055	0.022

## Discussion

This study’s main objectives were to quantify fatigue parameters in various trunk muscles during a lateral isometric hold test in healthy participants and to determine which primary trunk muscles are fatigued during this functional task. The ipsilateral external oblique, during both lateral isometric hold tests, and the contralateral L5 erector spinae, during the left lateral isometric hold test, showed significantly steeper negative NMF_slope_ than the other side’s respective muscles. The results also showed that all recorded trunk muscles presented fatigue parameters during lateral isometric hold tests on both sides. Thereby, these results provide a preliminary understanding of the trunk muscles tested during a lateral isometric hold task.

Even if the results showed a NMF_slope_ significantly different from 0 for the 8 muscles recorded, the percentage of decay did not exceed 0.07%s^-1^ which may raise questions with regard to the level of fatigue generated in individual muscles during the lateral hold. Despite the lack of normative data for fatigue parameters, other studies have reported steeper negative NMF_slope_ during trunk endurance protocols. Plamondon et al. [18] reported L3 erector spinae NMF_slope_ (%s^-1^ ± SD) of 0.10 ± 0.07 and 0.13 ± 0.05 for women and men respectively during modified Sorenson intermittent contraction tasks. Mannion et al. [16,17] reported NMF_slope_ values up to 0.46 ± 0.19 during modified Biering-Sorenson test. The results of the present study could suggest that muscles not recorded in our experiment could act as primary contributors during the lateral isometric hold task. In fact, the quadratus lumborum, which activity can be predicted with surface EMG placed on L3 erector spinae [15], has been reported to act as a trunk lateral flexor when contracted unilaterally [3,19], and some studies reported high levels of activity in this muscle during side bridges [11,20]. Lower limb muscle activity was not recorded in the present study, but gluteus medius, gluteus maximus and hamstring activation during different side bridges exercises has also been reported [6,12,13].

As often seen in motor tasks, there are certainly multiple muscle recruitment strategies (redundancy) that can be selected by the central nervous system to optimize task performance during a lateral isometric hold test [21]. During this task, more than one muscle contribute to the torque generated, and a changing combination of muscle forces to maintain the isometric contraction may be an efficient strategy to improve performance. Motor variability has been shown to be an efficient strategy to reduce the development of muscle fatigue [22]. Our results suggest that a variable trunk co-contraction strategy where numerous muscles contribute to the generation of isometric force is selected during a lateral isometric hold test. A few authors also reported global activation of trunk muscles instead of specific muscle recruitment during trunk endurance task. Page et al. [23] reported fatigue of various trunk muscles (i.e. abdominal muscles, lumbar erector spinae, biceps femoris) during sustained isometric contractions of abdominal muscles whereas Plamondon et al. [18] reported fatigue of lumbar erector spinae and hip extensor muscles (hamstring and gluteus maximus) during an intermittent modified Sorenson contraction task. Furthermore, other studies have reported the recruitment of several trunk muscles (e.g. quadratus lumborum, external oblique, rectus abdominis, lumbar and thoracis erector spinae, gluteus medius, lumbar multifidus, gluteus maximus and hamstrings) during side bridge tasks [11-13].

Our participants showed mean holding times similar to the ones reported in previous studies where lateral flexors endurance was evaluated using the side bridge position [8,9]. However, although only healthy participants were included in the study, a mean difference of more than 5% between both side holding times was observed. According to McGill, such differences in holding times during the side bridge position would suggest trunk muscle imbalance [10]. The relatively high variability of the right on left holding time ratio (i.e. a standard deviation of 11.8%) suggests a wide variation of this ratio in healthy populations. Differences in testing protocols (isometric lateral hold versus side bridge) may explain these differences. However, one might question the clinical value of the proposed criteria suggesting that differences of more than 5% between right and left holding times characterize individuals with a history of disabling back troubles or an increased risk of back trouble [10]. Others studies are necessary to validate the use of a holding time ratio derived from the lateral isometric hold test as a normative data to evaluate muscle balance and function.

A few limitations need to be considered when interpreting the present results. For this study, only healthy young adults were recruited, and consequently generalization to other healthy or clinical populations may be limited. Only eight trunk muscles (surface EMG) were recorded, and other studies are necessary to evaluate the possible contribution and fatigue of several other trunk or lower limb muscles that could be recruited during lateral isometric hold task.

## Conclusions

This study was conducted in order to identify muscles evaluated during a lateral isometric hold task. Although the fatigue indices suggest that ipsilateral external oblique and contralateral L5 erector spinae are significant contributors, all recorded muscles were active during the lateral isometric hold task. The lateral isometric hold, while different from the side bridge assessment for endurance because it does not involve support of the floor, seems to be adequate for the evaluation of lateral trunk flexors. Studies involving individuals with low back pain are needed to evaluate the clinical relevance of this procedure.

## Competing interests

The author(s) declare that they have no competing interests

## Authors’ contributions

IP participated in the study design, experimentation and manuscript writing. MD participated in study design, data analysis, manuscript writing and revision. All authors read and approved the final manuscript.
